# Substrate Recognition and Specificity of Chitin Deacetylases and Related Family 4 Carbohydrate Esterases

**DOI:** 10.3390/ijms19020412

**Published:** 2018-01-30

**Authors:** Hugo Aragunde, Xevi Biarnés, Antoni Planas

**Affiliations:** Laboratory of Biochemistry, Institut Químic de Sarrià, Universitat Ramon Llull, 08017 Barcelona, Spain; hugoaragunde@gmail.com (H.A.); xavier.biarnes@iqs.edu (X.B.)

**Keywords:** chitin deacetylases, carbohydrate esterases, chitosan, peptidoglycan, chitooligosaccharides, structure, substrate specificity, deacetylation pattern

## Abstract

Carbohydrate esterases family 4 (CE4 enzymes) includes chitin and peptidoglycan deacetylases, acetylxylan esterases, and poly-*N*-acetylglucosamine deacetylases that act on structural polysaccharides, altering their physicochemical properties, and participating in diverse biological functions. Chitin and peptidoglycan deacetylases are not only involved in cell wall morphogenesis and remodeling in fungi and bacteria, but they are also used by pathogenic microorganisms to evade host defense mechanisms. Likewise, biofilm formation in bacteria requires partial deacetylation of extracellular polysaccharides mediated by poly-*N*-acetylglucosamine deacetylases. Such biological functions make these enzymes attractive targets for drug design against pathogenic fungi and bacteria. On the other side, acetylxylan esterases deacetylate plant cell wall complex xylans to make them accessible to hydrolases, making them attractive biocatalysts for biomass utilization. CE4 family members are metal-dependent hydrolases. They are highly specific for their particular substrates, and show diverse modes of action, exhibiting either processive, multiple attack, or patterned deacetylation mechanisms. However, the determinants of substrate specificity remain poorly understood. Here, we review the current knowledge on the structure, activity, and specificity of CE4 enzymes, focusing on chitin deacetylases and related enzymes active on *N*-acetylglucosamine-containing oligo and polysaccharides.

## 1. Carbohydrate Esterases and the CE4 Family

Carbohydrate esterases (CEs) are enzymes that catalyze the de-*O*- or de-*N*-acetylation of glycans and substituted saccharides. To date (December 2017), there are 15 different CE families classified in the CAZY data base (Carbohydrate Active Enzymes, www.cazy.org) [1]. The substrates used by CE enzymes are very diverse, and the enzyme classes are named according to substrate preference: chitin deacetylases, peptidoglycan deacetylases, acetylxylan esterases, polysaccharide deacetylases, acetyl esterases, feruloyl esterases, pectin acetyl esterases, pectin methylesterases, glucuronoyl esterases, and enzymes catalyzing the de-*N*-acetylation of low molecular mass aminosugar derivatives [2,3,4,5].

The CE4 family is composed mainly of chitin deacetylases (CDAs) (EC 3.5.1.41) and chitooligosaccharide deacetylases (EC 3.5.1.-), peptidoglycan *N*-acetylglucosamine deacetylases (EC 3.5.1.104), peptidoglycan *N*-acetylmuramic acid deacetylases (EC 3.5.1.-), and poly-β-1,6-*N*-acetylglucosamine deacetylase (EC 3.5.1.-), though it also contains some acetylxylan esterases (EC 3.1.1.72) [6]. These enzymes share a conserved region known as the NodB homologous domain, due to its similarity to the NodB oligosaccharide deacetylase, one of the first deacetylases of this family to be characterized [7]. Deacetylases have been found in bacteria, fungi, and insects [3,8].

The first active CDA was identified and partially purified from extracts of the fungus *Mucor rouxii* [9]. Later, some active CDAs were identified and purified from very diverse organisms, such as archaea, marine bacteria, fungi, and insects, which in many cases, are not even capable of producing chitosans [10]. These enzymes, like their sources, are very diverse in their characteristics and optimal working conditions. Their molecular masses vary from 12.7 to 150 kDa, their isoelectric points (pIs) vary from 2.7 to 4.8, the optimum pH ranges from 4.5 to 12, and they show significant thermal stability, as their optimum temperatures for activity range from 30 to 60 °C. Most CDAs are highly inactive on crystalline chitin and have a preference for soluble chitins, such as glycol-chitin or chitin oligomers, as well as partially deacetylated chitin (chitosans). The inactivity on insoluble chitin is most likely due to the inaccessibility of the acetyl groups in the tightly packed chitin structure. Some CDAs contain carbohydrate binding modules (CBM) fused to the catalytic domain that seem to increase the accessibility of the chitin chains to the catalytic domain, resulting in a (slightly) enhanced deacetylase activity [11]. Recently, it has been shown that the addition of a lytic polysaccharide monooxygenase (LPMO), which oxidatively cleaves the chitin chains on the surface of the fibrils, greatly increased the activity of a CDA on β-chitin [12]. In terms of their cellular localization, CDAs are found extracellularly, in the periplasm or in the cytosol. In fungi, periplasmic CDAs are generally tightly coupled to a chitin synthase to rapidly deacetylate newly synthesized chitins before their maturation and crystallization. Extracellular CDAs are secreted to alter the physicochemical properties of the cell wall to either protect the cell wall from exogenous chitinases or to initiate autolysis. In bacteria, CDAs are either intracellular, as in *Rhizobium* species where they are involved in Nod factor biosynthesis, or extracellular, involved in the catabolism of chitin, as in marine bacteria [8,10,13].

## 2. Substrates of CE4 Family Enzymes

### 2.1. Chitin, Chitosan, and Their Oligomers

**Figure 1:**
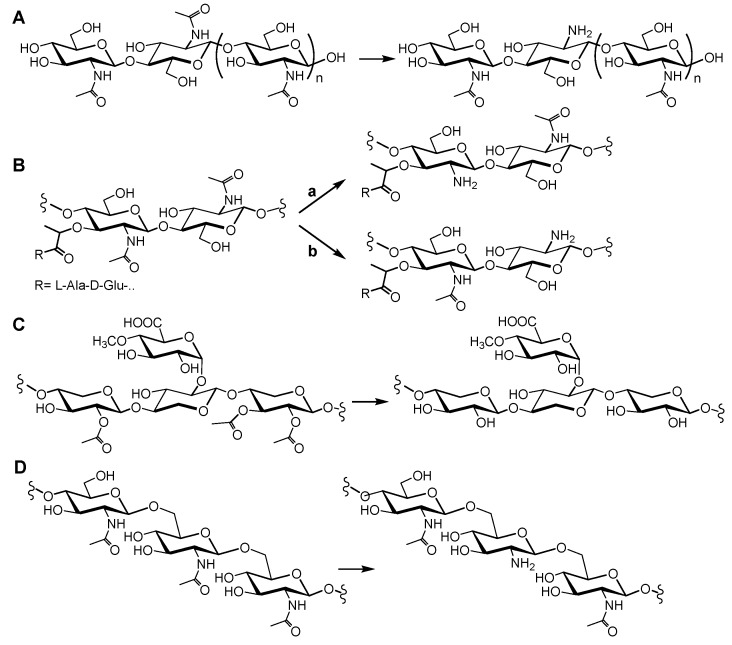
Structures of the substrates of CE4 enzymes and representative deacetylated products. (**A**) Chitin oligosaccharide substrate of chitin deacetylases; (**B**) Peptidoglycan fragment substrate of peptidoglycan MurNAc deacetylases (a) or peptidoglycan GlcNAc deacetylases (b); (**C**) Acetyl-d-glucurono-d-xylan substrate of acetylxylan esterases; (**D**) β-1,6-Glucan substrate of poly-β-1,6-*N*-acetylglucosamine deacetylases.

Chitin was first isolated from fungi by Braconnot in 1811 [14], and its structure was determined in 1929 by Hofmann [15]. Chitin is a linear polysaccharide of β(1→4)-linked *N*-acetylglucosamine monomers (Figure 1A). It is the most abundant natural amino polysaccharide, and it is also regarded as one of the most abundant molecules in nature after cellulose. Chitin is widely distributed, as a major structural component of the exoskeletons of arthropods (including insects and crustaceans), in the endoskeletons of mollusks (such as squid), and in the cell walls of fungi and diatoms [14,16,17]. Chitin is present as ordered macrofibrils, mainly in two allomorphs, α-chitin (the most abundant, usually isolated from the exoskeleton of crustaceans, particularly from shrimps and crabs) and β-chitin (extracted from squid pens), and additionally as γ-chitin, which appears to be a combination of the α and β structures (found in fungi and yeast) [18]. The processing of the chitin polymer in the form of depolymerization and de-*N*-acetylation reactions produces a series of new compounds, including chitosan and chitooligosaccharides. Chitosan is the result of de-*N*-acetylation of chitin. In nature, this reaction is almost never complete, meaning that chitosan is a generic name for heteropolymers composed of *N*-acetylglucosamine (GlcNAc) and glucosamine (GlcNH_2_). Only some fungi of the *Zygomycota*, *Basidiomycota*, and *Ascomycota* phyla have been reported to be capable of naturally producing chitosans [19]. The deacetylated units have free amino groups that, at slightly acidic conditions, convey positive charges to the polymers, making them the only known natural polycationic polysaccharides [14,16,19]. They interact with polyanionic biomolecules, such as proteins, nucleic acids, polyanionic phospholipidic membranes, and sulfated polysaccharides, like the human glycosaminoglycans at cell surfaces. Depolymerization of both chitin and chitosan yields their respective oligosaccharides [20]. Chitin and chitosans mainly act as structural polymers, while their oligomers are involved in molecular recognition events, such as cell signaling and morphogenesis, and act as immune response elicitors and host–pathogen mediators [21,22,23,24,25]. Hence, CDAs are candidates for the design of antifungals and antibacterials [8,10], and chitin derivatives have uses in medical, pharmaceutical, and cosmetic applications [26,27]. Chitosan polysaccharides and oligosaccharides are characterized by their degree of polymerization (DP), degree of acetylation (DA), and pattern of acetylation (PA).

### 2.2. Peptidoglycan

Peptidoglycan (PG) is a net-like molecule found in the cell wall surrounding the cytoplasmic membrane of almost all bacteria. It is a fundamental and specific structural element that helps preserve cell shape and protect cells against the internal osmotic pressure. As a result, any inhibition of its biosynthesis or its degradation during cell growth leads to cell lysis. However, it also serves as a scaffold for the attachment of specific proteins and other cell wall components [28,29,30,31]. Its chemical composition comprises long glycan chains crosslinked by short peptides, creating a large macromolecule. The glycan structure is a heteropolymer consisting of long chains of *N*-acetylglucosamine (GlcNAc) and *N*-acetylmuramic acid (MurNAc) residues linked β-1→4 in an alternating fashion (Figure 1B). The crosslinking peptide show some interesting characteristics, such as the presence of non-proteogenic aminoacids (e.g., diaminopimelic acid, lanthionine), alternating d- and l-isomers, and a γ-bonded d-glutamic acid. Peptidoglycan hydrolases have critical functions in peptidoglucan maturation, turnover, elongation, septation, and recycling, as well as in cell autolysis [32]. Post-synthetic modification of PG represents an important strategy for pathogenic bacteria to evade innate immunity and control autolysins. Modifications of the glycan backbone are generally restricted to the C-6 hydroxyl and C-2 amino moieties, with the most common being acetylation and deacetylation [33]. In particular, peptidoglycan *N*-deacetylases hydrolyze the amide linkage of the 2-*N*-acetyl groups of GlcNAc or MurNAc residues, some being active on complex peptidoglycan structures, but others having a preference for the saccharide chain after peptide release [33,34,35,36].

### 2.3. Acetylxylan

Acetylxylan is a plant polysaccharide that is a major component of the hemicellulose fraction of plant cell walls [37]. Like cellulose, most hemicelluloses function as supporting material in the cell wall [38]. Cellulose is entrapped in a hemicellulose matrix that includes several heteropolysaccharides formed by hexoses and pentoses. In hardwood trees, and some other annual plants, the main hemicellulose element is acetyl-d-glucurono-d-xylan (Figure 1C). Its backbone is formed by β-1,4-linked d-xylopyranosyl residues. Some of the xylose residues are α-1,2-substituted with 4-*O*-methyl-α-d-glucuronic acid, and almost every xylose residue is acetylated at positions 2 or 3, or both [38,39,40]. Acetyl xylan esterases de-*O*-acetylate the substituted xylosyl units with different specificities [4,40,41].

### 2.4. β-1,6-Glucan

Poly-β-1,6-*N*-acetyl-d-glucosamine (PNAG) (Figure 1D), also referred to as polysaccharide intercellular adhesin, is an exopolysaccharide that makes up the extracellular matrix of some bacterial biofilms [42]. Biofilm formation increases the survival of a colony during microbial infections by limiting the diffusion of antimicrobials and preventing phagocytosis [43]. Partial enzymatic deacetylation of PNAG is important for the maintenance of biofilms, since fully acetylated PNAG impedes biofilm formation [44]. Therefore, understanding the structure–activity relationships of PNAG deacetylases is relevant for the design of biofilm formation inhibitors [45,46].

## 3. CE4 Enzymes Active on Chitooligosaccharides and Their Substrate Specificities

Here, we focus on CE4 enzymes with characterized activity on chitooligosaccharides (COS) and/or a solved 3D structure by X-ray crystallography. These include CDAs (EC 3.5.1.41), as well as some peptidoglycan deacetylases and acetylxylan esterases for which activity on COS has been reported. Additionally, some poly-β-1,6-GlcNAc deacetylases, although with no reported activity on COS, are included because their substrate specificities and 3D structures are closely related to those of CDAs.

**Figure 2:**
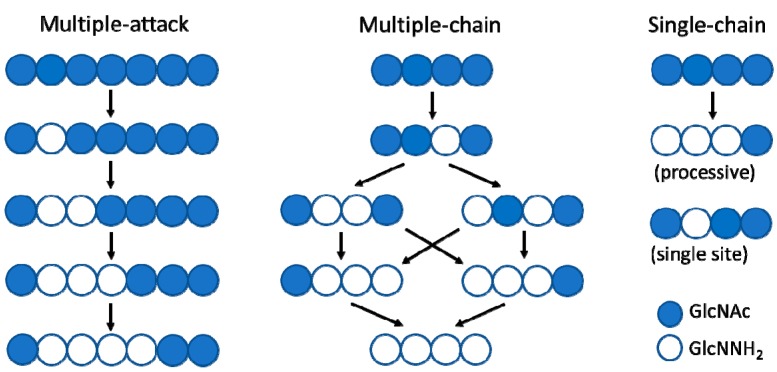
Modes of enzymatic action patterns for polysaccharide and oligosaccharide deacetylases: multiple-attack, multiple-chain, and single-chain mechanisms.

The deacetylation pattern exhibited by chitin deacetylases and related CE4 enzymes active on COS is diverse, reflecting different substrate specificities and pattern recognition on their linear substrates. Enzymatic action patterns for enzymes that modify in-chain units on a linear polysaccharide may be divided into three main types, designated multiple-attack, multiple-chain, and single-chain mechanisms (Figure 2) [3]. In the multiple-attack mechanism, binding of the enzyme to the polysaccharide chain is followed by a number of sequential deacetylations, after which the enzyme binds to another chain. (i.e., *M. rouxii* [47,48]). In the multiple-chain mechanism, the enzyme forms an active enzyme–polymer complex, and catalyzes the hydrolysis of only one acetyl group before it dissociates and forms a new active complex (i.e., *C. lindemuthianum* CDA [49,50]). Finally, a single-chain mechanism refers to processive enzymes in which a number of catalytic events occur on a single substrate molecule, leading to sequential deacetylation. This last group also includes the few CDAs with specificity for deacetylating a single position in chitooligosaccharide substrates (i.e., *Rhizobium* NodB or *Vibrio* CDA or COD, see below). While the multiple-chain mechanism with no preferred attack will result in a binary hetero-polysaccharide with a random distribution of the units, the multiple-attack and the single-chain mechanisms will generate block-copolymer structures.

A major challenge is understanding how these enzymes define the distribution of GlcNAc and GlcNH_2_ moieties in the oligomeric chain. Analysis of their substrate specificity in conjunction with their 3D structures and multiple sequence alignments provides the background of a structural model on the determinants of substrate specificity that dictates the deacetylation patterns. Table 1 summarizes the CE4 enzymes considered in this study. It includes chitin deacetylases that have been biochemically characterized with regard to their specificity on COS, and other CE4 family members with solved 3D structures (released on the PDB up to December 2017), such as peptidoglycan GlcNAc and MurNAc deacetylases, and acetylxylan esterases (some of them also active on COS), poly-β-1,6-GlcNAc de-*N*-acetylases (not active on COS), and putative polysaccharide deacetylases with reported 3D structures that either have unknown substrates or are inactive enzymes. The relevant information that has been reported on the substrate specificity of each enzyme listed in Table 1 is summarized below.

### 3.1. Chitin Deacetylases (CDAs)

#### 3.1.1. Fungal Chitin Deacetylases

Fungal CDAs are involved in fungal nutrition, morphogenesis, and development [3,8], participating in cell wall formation and integrity [51], in spore formation [52], germling adhesion [53], and fungal autolysis [54]. Pathogenic fungi must evade host immune responses to successfully penetrate and gain access to host tissues. Plants protect themselves by secreting chitinases that break the fungal cell wall chitin down to chitooligosaccharides (COS), which are recognized by plant chitin-specific receptors triggering resistance responses [55]. Plant fungal pathogens secrete CDAs during infection and the early growth phase in the host [8]. It has been hypothesized that fungi evade plant defense mechanisms by partially deacetylating either their exposed cell wall chitin or the chitooligosaccharides produced by the plant chitinases, hence, the resulting partially deacetylated oligomers are not further recognized by the specific receptors [55,56,57,58].

*Mucor rouxii CDA* (*Mr*CDA). The *Mucor rouxii* CDA was one of the first enzymes to be identified as a deacetylase. *M. rouxii* is a dimorphic fungus with a cell wall mainly composed of chitin, chitosan, and mucoric acid. *Mr*CDA is a specific enzyme for β-1,4-GlcNAc polymers, such as glycol-chitin, colloidal chitin, chitosan, and chitin; it also deacetylates acetylxylan, but it is inactive on peptidoglycan or acetyl heparin polymers [6,9]. It is active on chitooligosaccharides, and its activity increases with the degree of polymerization (DP) [9,59,60], with triacetylchitotriose being the smallest substrate it acts on [48]. It has been reported that the enzyme deacetylates its substrates following a multiple-attack mechanism [47], but the resulting pattern depends on the DP of the substrate: DP3, DP6, and DP7 substrates are not fully deacetylated, leaving the reducing GlcNAc unmodified [D_n−1_A], whereas DP4 and DP5 substrates are fully deacetylated. In all cases, deacetylation starts at the non-reducing end residue, and then proceeds to the neighboring monomer towards the reducing end [48].

*Colletotrichum lindemuthianum CDA* (*Cl*CDA). The plant pathogen deuteromycete fungus *C. lindemuthianum* is the causative agent of the anthracnose, which affects economically important crop species [78]. It secretes a CDA that is active on both chitin polymers (glycol-chitin) and chitin oligomers. It is able to fully deacetylate chitooligosaccharides with a DP equal to or greater than 3, while it only deacetylates the non-reducing GlcNAc of *N*,*N*′-diacetylchitobiose [49,79]. For substrates longer than DP3, it performs a multiple-chain mechanism, following a pathway in which the first residue to be deacetylated is the second from the reducing end [49,50]. The initial mono-deacetylation reaction shows no dependency of *k*_cat_ on DP, but *K*_M_ decreases with increasing DP [50,61]. However, when analyzing the full deacetylation kinetics, an increase in *k*_cat_ and reduction in *K_M_* correlates with an increase in substrate DP [80]. It has been reported that this enzyme is reversible, as it is also able to catalyze the acetylation of chitosan oligomers [81,82,83].

*Aspergillus nidulans CDA* (*An*CDA). *An*CDA is secreted into the extracellular medium to deacetylate the chitin oligomers produced by chitinases during cell autolysis [54,84,85,86]. The enzyme is active on soluble chitins (CM-chitin, glycol-chitin), colloidal chitin, chitosan, acetylxylan, and acetylated glucuronoxylan, but not on peptidoglycan [12,87]. *An*CDA is active towards chitooligosaccharides with a DP of 2 to 6 [12]. Long incubations with high enzyme concentration showed that the enzyme is inactive towards GlcNAc, and catalyzes the mono-deacetylation of (GlcNAc)_2_. For longer substrates, fully deacetylated products were produced. The deacetylation rate exhibits a counter-intuitive relationship with the length of the chitooligosaccharide substrates: odd-numbered chitooligosaccharides (DP5, DP3) have higher apparent rate constants than even-numbered oligomers (DP4, DP2). Monitoring of products formation with the DP6 substrate showed that the first deacetylation event occurs at random positions, except for the reducing end, which reacts much more slowly to yield the fully deacetylated product. With the DP5 substrate at short reaction times, the kinetic constants are *k_cat_* = 1.4 s^−1^ and *K_M_* = 72 μM, similar to those reported for *Cl*CDA.

*Puccinia graminis f.* sp. *Tritici CDA* (*Pgt*CDA). *Puccinia graminis f.* sp. *Tritici* is a biotrophic basidiomycete that is the causative agent of the stem rust [88]. *Pgt*CDA is active on polymers such as colloidal chitin and glycol-chitin, as well as on chitosans, where activity increases with the degree of acetylation. It is not active on insoluble polymers such as α- or β-chitin. With chitooligosaccharides, it was found that the minimal substrate is tetraacetylchitotetraose, as the enzyme is not able to act on shorter substrates. The sequence of the products obtained by enzymatic deacetylation of tetramers to hexamers reveals that the enzyme specifically deacetylates all but the last two GlcNAc units on the non-reducing end [AA(D)_n−2_] via a multiple-chain mechanism [89].

*Pestalotiopsis* sp. *CDA* (*Pes*CDA). *Pestalotiopsis* sp. is an endophytic fungus found in tropical areas that lives inside the tissues of its plant hosts [90]. PesCDA acts better on colloidal chitin as substrate, but it is also active on chitosans with a DA of 10–60% (higher activity with a higher DA), as well as on chitooligosaccharides [58]. It is not able to deacetylate crystalline chitin, neither α- or β-allomorphs. When analyzing the activity on oligomers, tetraacetylchitotetraose is the minimal substrate, but no substrate preferences or kinetic parameters have been reported for longer oligomers [48]. The enzyme follows a multiple-chain mechanism in which all residues are deacetylated, except the reducing end, and the last two GlcNAc residues from the non-reducing end, with a pattern of deacetylation [AA(D)_n−3_A] [58].

*Podospora anserina CDA* (*Pa*CDA). *Podospora anserina* is a filamentous ascomycete living as a saprophyte on herbivore dung [91]. *Pa*CDA has been recombinantly expressed in *Hansenula polymorpha* as a full length protein composed of the CE4 domain flanked by two CBM18 domains [11]. The enzyme is active on soluble glycol-chitin, chitosan polymers with a high DA, and chitooligosaccharides, and shows low activity on insoluble α- and β-chitin, which is reduced further by deletion of the CBM domains. On chitooligosaccharides, it is active against oligomers with a DP ≥ 2, leading to fully deacetylated products [D_n_]. The mode of action on DP3 and DP4 substrates revealed that it follows a multiple-chain mechanism. With the trimer, all possible isomers are found for both mono- and di-deacetylated intermediate products, although the first deacetylation event has a clear preference for the reducing end. This is not the case for the tetramer and pentamer substrates, where the residue next to the reducing end is preferentially deacetylated first, with the second deacetylation occurring mainly next to the existing GlcNH_2_ unit on either side. Overall, larger oligomers are deacetylated faster, with deacetylation of the reducing end occurring as a late event [11].

*Pochonia chlamydiosporia CDA (Pc*CDA). *Pochonia chlamydosporia* is a fungus belonging to the *Ascomycota* family that infects females and eggs of cyst or root-knot nematodes. It is used as a biocontrol agent against a number of plant parasitic nematodes in food-security crops [92,93,94]. *Pc*CDA deacetylates chitooligosaccharides, requiring at least four GlcNAc units in order to be active, but it prefers longer substrates. For DP4 and DP5 substrates, it first deacetylates the penultimate residue from the non-reducing end, and continues to the next residue towards the reducing end, with a pattern of acetylation [ADDA_n−3_] [95].

#### 3.1.2. Bacterial Chitin Deacetylases

*Sinorhizobium meliloti NodB* (NodB). Rhizobial NodB deacetylases were the first enzymes of the CE4 family to be described in detail [7,96,97]. NodB deacetylases are involved in the biosynthesis of Nod factors, the morphogenic signal molecules produced by rhizobia, which initiate the development of root nodules in leguminous plants [98]. NodB is active on chitooligosaccharides from DP2 to DP5 with no differences in *k_cat_*, but *K_M_* decreases with increasing DP. Specifically, *k_cat_*/*K_M_* is 5-fold higher for DP5 than for DP2 substrates [7,99]. DP4 or DP5 substrates are the natural substrates depending on the rhizobial strain. NodB only deacetylates the non-reducing end residue [DA_n−1_] [7,100,101], but traces of a second deacetylation event were seen upon long incubations [100].

*Vibrio cholera CDA* (*Vc*CDA or COD). Chitin oligosaccharide deacetylases (COD) from the *Vibrionaceae* family are involved in the chitin degradation cascades occurring in sea water [102,103,104,105]. They have been identified in many *Vibrio* species, such as *V. algynolyticus* [106,107], *V. parahaemolyticus* [108,109], *V. cholera* [110], and others. Whereas *V. parahaemolyticus* or the *Vibrio* sp. SN184 only deacetylate DP2 and DP3 substrates, the *Vibrio cholera* chitin deacetylase (*Vc*CDA) has a broader specificity, being active on DP2 to DP6 substrates [62,110]. *Vc*CDA is 10-fold more active on DP2 than DP4 substrates [62], and it is highly specific for deacetylation of the penultimate residue from the non-reducing end, generating monodeacetylated products with the pattern [ADA_n−2_] [62,100,110].

*Arthrobacter* sp. *CDA* (*Ar*CE4). Arthrobacter species are Gram-positive bacteria known to grow on chitin and secrete chitinases [111,112,113]. ArCE4 was identified from a search of monodomain and extracellular deacetylases in annotated genomes and metagenomes [64]. *Ar*CE4 is active on α- and β-chitin, chitosan (DA 64%), and acetylxylan. On COS substrates, activity increases with increasing DP, with higher activity against DP5 compared to DP6, and no activity on GlcNAc. The enzyme acts by a multiple-chain mechanism, as shown with the DP5 substrate, where different mono- and di-deacetylated products were obtained. The first deacelylation happens at all three internal positions, whereas di-deacetylation mainly occurs at the GlcNAc unit next to the reducing end, and at either of the two other internal units (ADDAA and ADADA). Although other minor products are formed, it seems that the reducing end unit is not deacetylated [64].

### 3.2. Peptidoglycan Deacetylases

#### 3.2.1. GlcNAc Peptidoglycan Deacetylases

*Streptococcus pneumoniae Sp*PgdA. *S. pneumoniae* is a Gram-positive bacteria, and one of the most important human pathogens, responsible for pneumonia, otitis media, and meningitis [114]. Deacetylation of the peptidoglycan is used as a defense mechanism, reducing the likelihood of being hydrolyzed by lysozyme [26]. On peptidoglycan, *Sp*PgdA deacetylates up 84% of the GlcNAc residues, but also deacetylates MurNAc residues to a lesser extent (10% of the total) [35]. It can also deacetylate chitin oligomers, having been tested so far on triacetylchitotriose and pentaacetylchitopentaose, and being inactive on GlcNAc [65,115]. Only the central residue of the DP3 substrate is deacetylated [ADA] [65]. With the DP5 substrate, mono-, di-, and tri-deacetylated chitosan oligomers are obtained, but the deacetylation pattern has not been reported [115]. Interestingly, it is not capable of using soluble low molecular weight oligomers of its own peptidoglycan or peptidoglycan from other species.

*Streptococcus mutans Sm*PgdA. *SmPgdA* is involved in protecting this pathogenic bacteria from the innate immune system [66]. *SmP*gdA seems to have no activity on peptidoglycan, and its natural substrate is yet unidentified [66]. However, it exhibits de-*N*-acetylase activity towards hexaacetylchitohexaose. No activity was observed with shorter chitooligosaccharides or a synthetic peptidoglycan tetrasaccharide.

*Bacillus cereus Pgd BC1960* (*Bc*Pgd). *Bacillus cereus* is a Gram-positive bacteria found in soil and food that can cause diarrhea in humans [116]. The genome of *B. cereus* contains eleven ORFs for putative polysaccharide deacetylases, five of which have been identified as peptidoglycan GlcNAc deacetylases. BC1960 (*Bc*Pgd) is active on peptidoglycan, but partially hydrolyzed peptidoglycan is a better substrate than the native form. It has been found that up to 85% of the GlcNAc residues are modified by the enzyme [117]. Small peptidoglycan fragments are not substrates. It is also able to deacetylate peptidoglycans from other species, and it is not active on xylan or acetyl-heparin. *Bc*Pgd deacetylates soluble chitin and chitooligosaccharides. In terms of *k_cat_*, the order of preference for chitooligosaccharides is DP4 > DP3 > DP2 > DP5 > DP6, whereas *K_M_* decreases with DP [118]. The pattern of acetylation of the products [D_n−1_A] indicates that the enzyme deacetylates all the residues but the reducing end, although fully deacetylated products are observed with DP3 and DP4 substrates upon long incubation times. Other *B. cereus* peptidoglycan deacetylases have also shown promiscuous substrate specificity, being active on peptidoglycan, glycol-chitin, and chitooligosaccharides. BC3618 has a kinetic behavior similar to *Bc*Pgd (BC1960) on chitooligosaccharides, and it does not deacetylate the reducing end GlcNAc, with final products having the pattern [D_n−1_A] [118]. Likewise, BC1974, BC2929, and BC5204 deacetylate hexaacetylchitohexaose with different deacetylation patterns, but do not deacetylate the reducing and non-reducing terminal GlcNAc residues of the oligosaccharide [119].

*Eubacterium rectale Pgd (Er*Pgd). *Eubacterium rectale* is part of the adult human distal gut microbial community, and belongs to Clostridium Cluster XIVa, one of the most common gut *Firmicutes* clades [120]. The 3D structure of a predicted peptidoglycan GlcNAc deacetylase has been solved, but its biochemical characterization has not yet been reported. Although it should be included in the “unknown” subclass in Table 1, it is placed in the group of characterized peptidoglycan GlcNAc deacetylases, due to its high similarity and active site motif conservation with other subclass members, as discussed below.

#### 3.2.2. MurNAc Peptidoglycan Deacetylases

*Bacillus subtilis PdaA (Bs*PdaA). *Bacillus subtilis* is a Gram-positive bacteria found in soil and the gastrointestinal tract of some animals, and has the ability to form spores to survive harsh environmental conditions [121]. *Bs*PdaA is involved in autolysis during spore germination [122]. It deacetylates MurNAc residues of the glycan chain after peptide removal from the peptidoglycan by Cwld (an l-alanine amidase) [123]. The deacetylated MurNH_2_ is a precursor for muramic δ-lactam synthesis, which involves transpeptidase activity. Introduction of the *pdaA* and *cwlD* genes into *E. coli* cells led to lactam formation in peptidoglycan, which suggests that PdaA participates in both steps, the de-*N*-acetylation of muramic acid residues, and the transpeptidase reaction for lactam cyclization [124]. However, no transpeptidase activity was detected in vitro, where PdaA only deacetylates *N*-acetylmuramic acid residues without peptide side chains. It does not recognize native peptidoglycan, soluble chitin, chitin oligomers, or GlcNAc as substrates [123].

*Bacillus anthracis Pda* (*Ba*Pda). *B. anthacis* is a Gram-positive spore forming bacteria that is the causative agent of anthrax [125]. Like its close relative *B. cereus*, its genome contains ten ORFs for putative CE4 deacetylases, with more than 90% identity to the corresponding orthologues in *B. cereus*, including GlcNAc and MurNAc deacetylases [118]. In particular, *Ba*Pda (BA0424) has been crystallized [69], and it is closely related to *B. subtilis* PdaA, thus suggesting a similar substrate specificity on MurNAc residues of the peptidoglycan polymer.

### 3.3. Putative Polysaccharide Deacetylases (PPda)

Four enzymes in Table 1 with solved 3D structure have unknown substrates or are inactive enzymes.

*Bacillus cereus BC0361*. Of the eleven ORFs encoding for putative polysaccharide deacetylases, five have been experimentally proven to be active on peptidoglycan, as discussed above. BC0361 has been crystallized, and its 3D structure has been solved [70]; it is predicted to be a peptidoglycan GlcNAc deacetylase for which the substrate remains unknown.

*Bacillus anthracis BA0330.* BA0330 and BA0331 are the only lipoproteins among the eleven known or putative polysaccharide deacetylases of *B. anthracis*. The 3D structure of BA0330 has been solved. The enzyme is not active against glycol-chitin, chitooligosaccharides, synthetic muropeptide, or *p*-nitrophenyl acetate [71]. It maintains the conserved metal-coordination motif and catalytic residues, but with some arrangements and alterations that will be discussed later.

*Bacillus anthracis BA0150*. Another putative Pda from *B. anthracis* (*Ba*PdaB, BA0150) has been crystallized [72]. However, the crystal structure does not contain a catalytic metal ion, and the protein does not contain the conserved Asp–His–His metal-binding triad that is found in most of the CE4 enzymes, indicating that it is not a functional polysaccharide deacetylase. However, since it contains the conserved Asp and His catalytic residues, BA0150 may have some other hydrolytic activity [72].

*Encephalitozoon cuniculi ECU11_0510*. The microsporidian *E. cuniculi* is an intracellular eukaryotic parasite [126,127]. ECU11_0510 was annotated as a putative polysaccharide deacetylase but was found to be unable to deacetylate chitooligosaccharides or crystalline β-chitin. It lacks the conserved aspartic residue, acting as catalytic base in all CE4 family members. It has been speculated that it is an inactive enzyme that evolved from a former chitin deacetylase, since the organism has developed an infectious mechanism that may require rigidity of the infectious spore which is enhanced by the presence of chitin and that would be disrupted by chitosan production [73].

### 3.4. Acetylxylan Esterases

*Streptomyces lividans AXE* (*Sl*AxeA). *Streptomyces lividans* is a Gram-positive bacteria known to secrete large quantities of proteins involved in the catabolism of plant cell walls [128]. Acetylxylan esterases are an important part of the xylanolytic enzyme system [129,130,131], since they deacetylate acetylglucuronoxylans and acetylglucuronoarabinoxylans, making them accessible to endo-xylanases. *Sl*AxeA can deacetylate both 2 and 3 *O*-acetylated positions of xylose, but shows a preference for the 2 position. Its activity on double substituted xyloses is very slow, due to the requirement of a free OH in the 2 or 3 position. After the first deacetylation event, the second one occurs orders of magnitude faster [132,133,134]. The enzyme also catalyzes the hydrolysis of *N*-acetyl groups in chitinous materials of variable degrees of polymerization and acetylation, such as glycol-chitin, chitosans with low DAs, and chitooligosaccharides [135]. The activities of wild type *Sl*AxeA and mutants (obtained by random mutagenesis) on hexaacetylchitohexaose reveals a multiple-chain mechanism, generating a mixture of mono- to pentadeacetylated products. Full deacetylation was not observed but, interestingly, some partially deacetylated products are deacetylated at the reducing end [136]. Additionally, it seems to be more active on chitin oligomers than on long polysaccharides [6]. The addition of Co^2+^ has a larger activating effect on chitinous substrates than on xylan substrates, with a 7-fold increase in activity on *N*,*N*′-diacetylchitobiose [6,137].

*Clostridium thermocellum AXE* (*Ct*AxeA). *C. thermocellum* is a Gram-positive bacteria known for its efficient plant cell wall degradation capabilities through a cell-bound multi-enzyme complex known as cellulosome [138,139]. One of these enzymes is the Xyn11A, which comprises a complex multi-domain structure, including a GH11 endoxylanase domain followed by a family 6 CBM, a dockerin domain, and the typical CE4 deacetylase domain (*Ct*AxeA) [140]. The *Ct*Axe module [74] is active on acetylxylans with Co^2+^ as the preferred cation [140,141]. No activity has been reported on chitinous substrates.

### 3.5. Poly-β-1,6-GlcNAc de-N-acetylase

Bacterial poly-β-1,6-GlcNAc de-*N*-acetylases are involved in biofilm formation, and are targets for the design of inhibitors [46,142,143,144,145,146]. The X-ray structure of four poly-β-1,6-GlcNAc de-*N*-acetylases have been reported (Table 1). Although not active on β-1,4-linked GlcNAc substrates, their structures and active site topology provide additional insights into the substrate specificities of the CE4 enzyme family.

*Escherichia coli* PgaB (*Ec*PgaB) deacetylates 3–20% of the GlcNAc residues of the PNAG, and facilitates its transport through the periplasmic space to the extracellular matrix. *Ec*PgaB specifically deacetylates GlcNAc polymers linked through β-1,6-linkages, and it is not able to use β-1,4-chitinous substrates [46]. Its activity increases with the DP of the substrate up to the hexamer. On the tetramer, deacetylation occurs at the second or third position from the non-reducing end, whereas on the pentamer, it first deacetylates the central GlcNAc residue, followed by the next residue on the reducing end side to generate a di-deacetylated product [75,76]. As will be discussed later, *Ec*PgaB has some variation in the positioning of the conserved CE4 catalytic residues, which explains the low efficiency of the enzyme, having a *k_cat_*/*K_M_* of 0.25 M^−1^s^−1^ for the β-1,6-pentasaccharide substrate [46].

Other poly-β-1,6-GlcNAc de-*N*-acetylases with solved X-ray structures, so far, are *Ad*IcaB from *Ammoniex degensii* [77], *Bb*BpsB from *Bordetella bronchiseptica* [45], and *Aa*PgaB from *Aggregatibacter actinomycetemcomitans* [147]. All of these enzymes show low activity on β-1,6-glucan, a common property of this enzyme subclass within family CE4.

## 4. Structural and Sequence Features of CE4 Enzymes Active on Chitooligosaccharides

### 4.1. Domain Organization

Some CE4 enzymes are monodomain proteins, but some others have a multidomain architecture composed of the CE4 catalytic domain (referred as the NodB homology domain) and several other domains, including carbohydrate binding modules (CBMs) and domains with unknown function. The function of CBMs is to facilitate the solubilization of substrates and enzyme–substrate recognition, but they are also involved in protein localization by concentrating the appended enzyme on to the polysaccharide substrate [148]. Table 2 summarizes the domain organization of the CE4 enzymes listed in Table 1 as predicted by Interpro, with the Uniprot accession codes.

### 4.2. X-ray Structures

**Figure 3:**
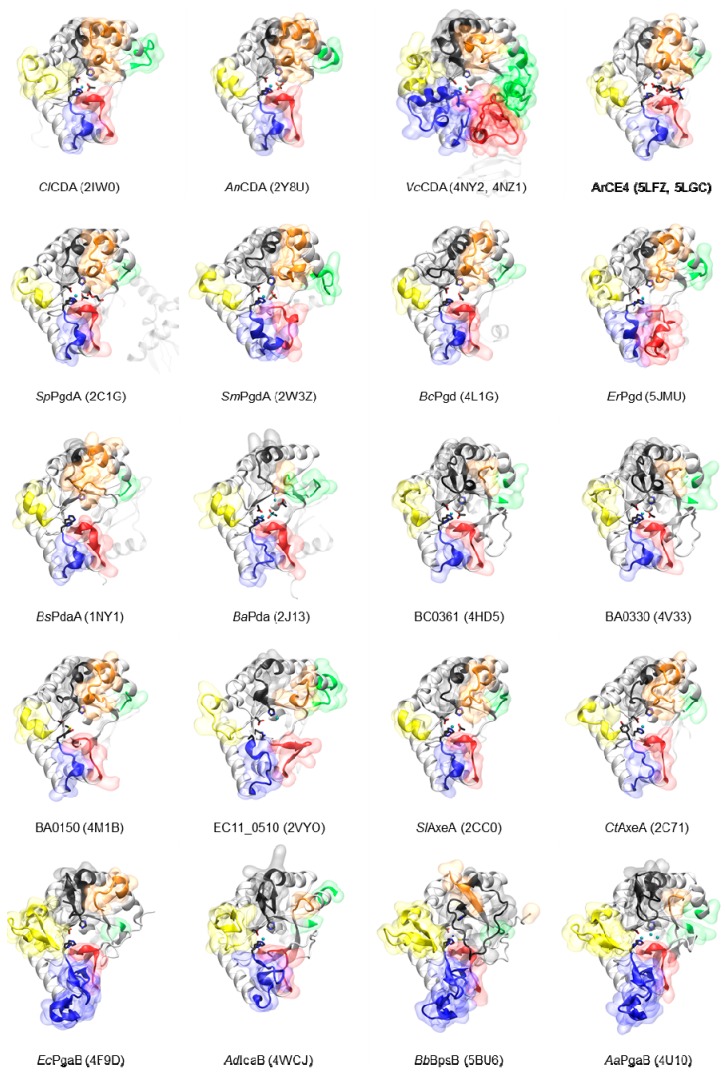
Three-dimensional structures by X-ray crystallography of the CE4 enzymes listed in Table 1. The *Vp*CDA structure (3WX7) is essentially identical to that of *Vc*CDA. Loops are colored as in Figure 4.

The CE4 enzyme members with solved 3D structure by X-ray crystallography are listed in Table 1, and their 3D structures are presented in Figure 3. The first structure of the CE4 family was that of *Bacillus subtilis* peptidoglycan deacetylase (*Bs*PdaA) in 2004 (PDB 1W17) [68]. After that, the structure of *Streptococcus pneumoniae* peptidoglycan GlcNAc deacetylase (*Sp*PgdA) was solved at 1.75 Å resolution (PDB 2C1G) and unraveled that the enzyme, and by extension other CE4 members, are metalloenzymes using a His–His–Asp zinc-binding triad [65]. The first structure of a CDA was that of *Choletotrichum lindemuthianum* in 2006 [61], which also showed that the enzyme employs a conserved His–His–Asp zinc-binding triad, closely associated with the conserved catalytic base (aspartic acid) and acid (histidine), to carry out acid/base catalysis. The *Aspergillus nidulans* CDA structure also reinforced the structural conservation of active site residues in chitin deacetylases [12].

The structure of the CE4 domain is characterized by an (β/α)_8_ barrel fold, which is frequently distorted, and may even appear as an (β/α)_7_ barrel that lacks one of the αβ repeats of regular TIM barrels, which creates a groove into which the extended polymer substrate binds [2,65,76]. The central core comprises seven or eight parallel β-strands that form a greatly distorted β-barrel surrounded by α-helices. A series of loops decorate the β-barrel and make up the majority of the carbohydrate binding pocket. There are significant topological differences among CE4 enzymes, with some having the N/C-termini on the same side of the barrel (as in *Cl*CDA), whereas in others, the N/C-termini are located on opposite ends (as in *Sp*PgdA and *Bs*PdaA). So far, all the structurally defined members of the CE4 family adopt a degree of “secondary structure swapping” from the canonical (β/α)_8_ fold [61].

A few other CE4 enzyme members were subsequently crystallized in their unliganded forms (Table 1, Figure 3). Acetylxylan esterases from *S. lividans* and *C. thermocelum* [74] show the canonical active topology and metal coordination in this enzyme family, as well as other peptidoglycan deacetylases (*S. mutants* [74], *B. anthracis* [69], and *B. cereus* [150]). The case of poly-β-1,6-GlcNAc deacetylases is different [75,76,77,147], as they have a circularly permutated CE4 domain and some variations in the positioning of active site residues, as discussed below. It was not until 2014 that the first crystal structure of a CE4 enzyme in complex with substrates was solved, that of the *Vibrio cholera* CDA [62], providing new information on loop organization and insights into the structural determinants of substrate binding, specificity, and catalysis.

### 4.3. Multiple Sequence Alignment of the CE4 Domain

**Figure 4:**
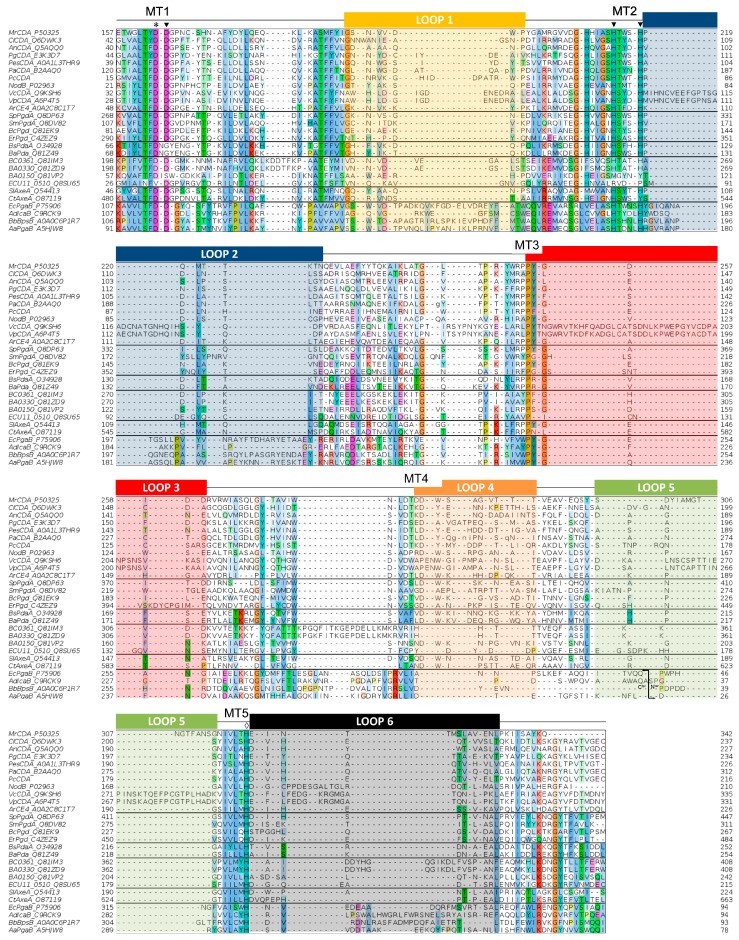
Multiple sequence alignment of the CE4 enzymes listed in Table 1. Loops are highlighted with colored boxes according to [62]. Conserved catalytic motifs are labelled MT1–5. The “His–His–Asp” metal binding triad (▼), catalytic base (*), and catalytic acid (◊) are labelled. The mark inside Loop 5 for poly-β-1,6-GlcNAc deacetylases (four last sequences) indicates the shuffling point of the circularly permuted CE4 domain.

The multiple sequence alignment of the CE4 domain of the enzymes reported in Table 1 is presented in Figure 4. Since standard multiple sequence alignment algorithms (based on sequence composition only) fail to reproduce the structural conservation of CE4 enzymes, the alignment was guided by the structural superposition of the available X-ray structures of these enzymes. This ensures that the alignment reproduces the conserved spatial distribution of amino acids, which is important for deciphering the structure–function relationships detailed in the following sections. Sequences of enzymes without structural data were incorporated into the alignment by means of Hidden Markov Model (HMM) comparisons. The 3D structures of *Vc*CDA and *Ec*PgaB were key to obtaining a complete multiple sequence alignment that spanned the whole CE4 domain. On one hand, the *Vc*CDA sequence exhibits substantially longer insertions than most of the other CE4 members do. On the other hand, the *Ec*PgaB sequence is shuffled around the C-terminus, which is a specific feature of the poly-β-1,6-GlcNAc deacetylase subfamily within the CE4 family (a circularly permuted CE4 domain). The shuffling point of *Ec*PgaB and related enzymes is indicated with an arrow in Figure 4. The multiple sequence alignment reveals an even distribution of conserved motifs and non-conserved insertions along the sequences of CE4 enzymes. Conserved motifs related to enzymatic activity are numbered from Motif 1 to Motif 5 (detailed in the next section). These are typically located at the center of the active site structure. Insertions are of variable length and variable sequence composition throughout the family. This variability decreases when considering only CE4 subfamilies with concrete enzymatic specificity. These insertions correspond to both unstructured and structured loops of variable geometry that surround the active site. These loops are numbered from Loop 1 to Loop 6 (Figure 4), and their influence in determining the substrate specificity of CE4 enzymes is discussed in the last section of this review.

### 4.4. NodB Domain and Active Site Conserved Motifs

CE4 family members share the NodB homology domain, which is approximately 150 aa long. This region is generally defined by five conserved motifs, named Motif 1 to Motif 5 according to the order they appear in the sequence. These consensus motifs were first proposed after the 3D structure of *Sp*PgdA was solved in 2005, in combination with a multiple sequence alignment of representative members of the CE4 family [65]. These motifs form the active site, and are required for enzymatic activity. As new 3D structures have been reported, the description of the conserved motifs can be refined based on more extensive sequence and structural alignments.

**Figure 5:**
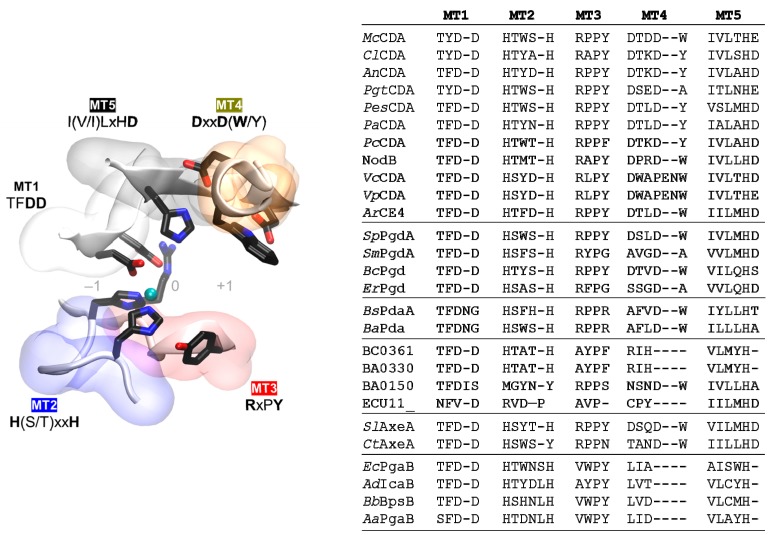
Conserved catalytic motifs MT1–5 of the CE4 family. (**Left**) Spatial disposition in the 3D active site structure; (**Right**) Motif sequences for the enzymes listed in Table 1. Subfamilies separated by a line: CDAs, peptidoglycan GlcNAc deacetylases, peptidoglycan MurNAc deacetylases, unknown, acetylxylan esterases, and poly-β-1,6-GlcNAc deacetylases.

Active site conserved motifs are highlighted in Figure 4 and shown in Figure 5. Motif 1 (TFDD) includes the general base aspartate (first D) and the metal-binding aspartate (second D). Motif 2 contains the consensus sequence H(S/T)xxH, which is regarded as a zinc-binding motif, where the two His residues bind the metal cation and the Ser or Thr residue forms a hydrogen bond with the second His, stabilizing the local conformation of the loop-shaped motif. The metal-binding Asp from Motif 1, plus the two His residues in Motif 2, are often designated the His–His–Asp metal-binding triad of CE4 enzymes. Motif 3 (RxPY) forms one of the sides of the active site groove, and establishes stabilizing interactions with other active site residues. Motif 4 (DxxD(W/Y)) forms the other side of the active site groove, including a hydrophobic residue exposed to the solvent and a buried Asp. Motif 5 is defined by I(V/I)LxHD, which contains a Leu as part of the hydrophobic pocket that accommodates the acetate methyl group of the substrate and the general acid His residue for catalysis.

## 5. Substrate Recognition and Catalysis

### 5.1. Catalytic Mechanism

**Figure 6:**
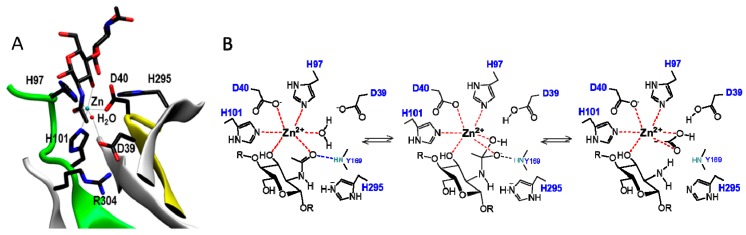
(**A**) Active site residues in the X-ray structure of the *Vc*CDA·DP2 complex, showing Zn^2+^ coordination and substrate binding; (**B**) Metal-assisted general acid/base mechanism proposed for CE4 deacetylases. Scheme based on the 3D structure of the enzyme–substrate complex *Vc*CDA_D39S_·DP2 [62]. D39 is the general base and His295 is the general acid.

CDAs and related CE4 enzymes operate by metal-assisted acid/base catalysis, analogous to other metal-dependent hydrolases [151]. The first CE4 structure solved (*Bs*PdaA [68]) was not conclusive regarding metal binding, but the subsequent structures of *Sp*PgdA [65] and xylan esterases [74] supported the proposed mechanism of metal-dependent CE4 enzymes. *Cl*CDA was also shown to contain a zinc cation in the active site, even though the enzyme was not inhibited by the metal chelator EDTA, indicating that the metal cation is tightly bound to the enzyme active site [61]. Further support arrived with the 3D structure of *Vc*CDA in complex with substrates [62]. The structure of complexes of an inactive mutant (at the general base Asp residue) with *N,N′*-diacetylchitobiose (DP2) and *N*,*N*′,*N*′′-triacetylchitotriose (DP3) in productive binding for catalysis showed that a sugar hydroxyl group of the substrate also participates in metal coordination. Specifically (Figure 6A), the carboxylate group of Asp40 and the imidazole rings of His97 and His101 are involved in Zn^2+^ coordination, which is also coordinated to the O7 atom of the *N*-acetyl group and O3 hydroxyl of the GlcNAc ring. A water molecule completes the distorted octahedral coordination to the divalent metal cation. Upon activation, this water molecule is proposed to be the nucleophile responsible for removal of the *N*-acetyl group [62].

The consensus mechanism of CDAs and related CE4 deacetylases is shown in Figure 6B. The carbonyl amide of the substrate is coordinated with the metal cation, and catalysis proceeds by nucleophilic attach of a water molecule activated by the general base (Asp) leading to a tetrahedral oxyanion intermediate. This oxyanion is stabilized by the metal and other active site residues. Protonation of the nitrogen group of the intermediate by the general acid (His) then facilitates C–N bond breaking with the generation of a free amine in the de-*N*-acetylated product, and release of acetate.

Hammett linear free energy correlations using α-haloacetamido substrate analogues performed on the *Cl*CDA enzyme provided further kinetic evidence of the presence of an oxyanion tetrahedral intermediate, and significant negative charge development at the transition state [61]. This charge would be stabilized by the oxyanion hole generated by the Tyr backbone nitrogen (Y145 in *Cl*CDA, Y169 in *Vc*CDA, shown in Figure 6) and the zinc cation.

The general acid and base residues are part of two conserved “charge relay” side chain pairs consisting of the catalytic base (Asp) tethered by a conserved Arg (in MT3, **R**xxPY), and the catalytic acid (His) tethered by a conserved Asp (in MT4, Dxx**D**(W/Y)), which may contribute to tuning the p*K*_a_ of the catalytic residues [61,65].

Although the conserved motifs MT1–5 are a signature for the family, close inspection of the positioning of active site residues within the five conserved motifs in the sequence alignment or in the 3D structures reveals different special arrangements that seem (tentatively, given the reduced number of proteins with experimentally proven specificity (Table 1)) to be characteristic of each enzyme subfamily (Figure 5).

Motif 1 (TFDD, with Y substituted for F in a few cases) is highly conserved within all CE4 members, except for peptidoglycan MurNAc deacetylases. They lack the Asp residue involved in metal coordination, and have an Asn insertion that points away from the metal ion into the core of the protein (*Bs*PdaA [68], *Ba*Pda [69]). The *E. coli* protein ECU11_0510 lacks the general acid Asp, and consequently, it is an inactive protein with unknown function [73]. In MT2 (H(S/T)xxH), poly-β-1,6-GlcNAc deacetylases have four residues separating the two metal binding His residues, as opposed to three in the other subfamilies. Significantly, inactive proteins classified as “unknown” in Table 1 lack these conserved His residues. The MT3 motif is well conserved, except for poly-β-1,6-GlcNAc deacetylases, where the Arg residue interacting with the general base in the other CE4 members (**R**xPY) occurs further in the sequence just before MT4 [75]. Poly-β-1,6-GlcNAc deacetylases also differ in MT4; they have a water molecule in place of the conserved Asp that interacts with (and presumably activates) the catalytic acid His in the other CE4 members. It has been hypothesized that this water molecule would not be able to activate the catalytic acid in the same manner that an aspartic acid residue would, which is consistent with the lower specific activity of these enzymes relative to the other CE4 subfamilies [75]. Finally, the general acid His in MT5 is strictly conserved. These motifs also play important roles in substrate recognition, and these will be described below.

### 5.2. Substrate Recognition and Specificity

#### 5.2.1. The Case of *Vc*CDA: Enzyme·Substrate Complexes Show an Induced Fit Mechanism

**Figure 7:**
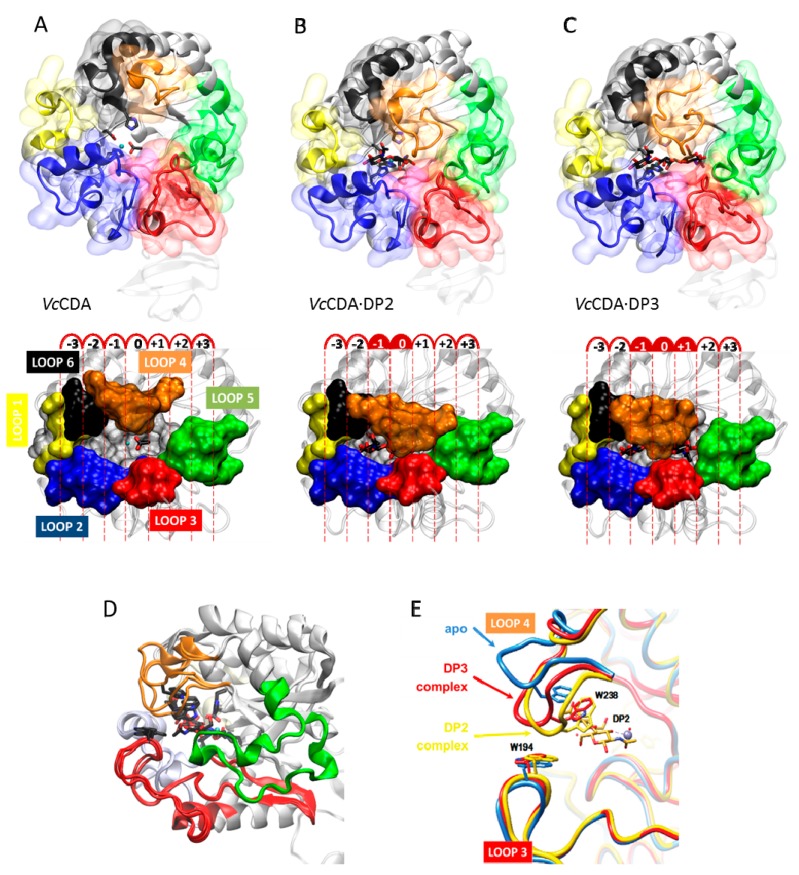
Crystallographic structure of (**A**) *Vc*CDA in the unliganded form (free enzyme with Zn^2+^ and acetate); (**B**) Binary complexes with DP2; and (**C**) DP3 ligands; (**D**) Superimposition of the three structures. Loop 4 (brown) has different conformations; (**E**) Magnification of the active site Loop 4 in the unliganded form (blue), and in enzyme–substrate complexes with DP2 (yellow) and DP3 (red) ligands. Only the DP2 ligand is shown.

The series of crystal structures of *Vibrio cholera*e chitin oligosaccharide deacetylase reported in 2014 [62] were the first 3D structures of a CE4 enzyme in complex with substrates. These new data paved the way for deciphering detailed structure–function relationships for this family of enzymes. Currently available structures of *Vc*CDA include the unliganded form of the enzyme, and the binary complexes with *N*-acetyl-glucosamine (DP1), *N*,*N*′-diacetylchitobiose (DP2), and *N,N*′*,N*′′-triacetylchitotriose (DP3), (Figure 7A). Both the DP2 and DP3 complexes revealed the exact location of the substrate in the active site of the enzyme in a competent binding mode for catalysis. These structures provided further evidence for the proposed metal-assisted acid/base catalytic mechanism, and revealed key enzyme–substrate interactions, along the catalytic itinerary (discussed in the previous section, Figure 6).

Remarkably, the binding cavity at the catalytic center is dynamically assembled upon substrates binding (DP ≥ 2): important conformational changes in a loop in the structure (namely, Loop 4, see later) take place induced by the binding of different substrates. The structure of this loop alternates between an open conformation in the unliganded *Vc*CDA and *Vc*CDA·DP1 complex, and a completely closed conformation in the *Vc*CDA·DP2 complex, or a semi-closed conformation in the *Vc*CDA·DP3 complex (Figure 7B). Such induced-fit conformational changes upon substrate binding are triggered by the CH–π stacking interaction [152,153] established between Trp238 (located in Loop4, MT4 motif DxxD(**W**/Y)), and the *N*-acetylglucosamine unit at the catalytic center. Since either a tryptophan or tyrosine residue is predominantly observed in the equivalent position in other CE4 structures (Figure 5), it is reasonable to think that similar induced-fit conformational changes may occur in other CE4 enzymes as well.

#### 5.2.2. Determinants of Substrate Specificity: The Subsite Capping Model

**Figure 8:**
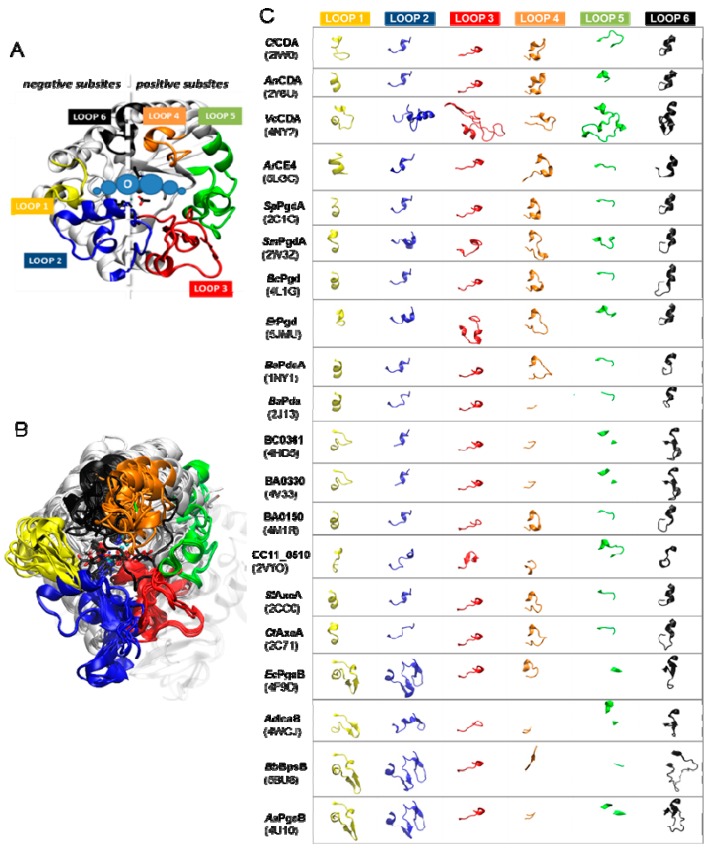
(**A**) *Vc*CDA structure with labelled loops 1 to 6. Loops 1, 2, and 6 shape the non-reducing end (negatives) subsites, and Loops 3, 4, and 6 define the reducing end (positives) subsites; (**B**) Superposition of all 3D structures of CE4 enzymes with solved X-ray structure (Table 1). The core of the proteins (in grey) is highly conserved, and main differences are on the loops surrounding the binding site cleft. Loops colored as in A; (**C**) Comparison of topology of Loops 1 to 6 for the enzymes overlaid in B.

The diversity of deacetylation patterns exhibited by chitin deacetylases and related CE4 enzymes active on COS (Table 1) can be attributed to the differential accessibility of the linear chitin oligosaccharide to the separate subsites along the substrate binding cleft of their structures. The ability of the substrate to slide along the binding cleft or to bind in different modes will determine which *N*-acetylglucosamine units can be exposed to the catalytic site, where the actual deacetylation reaction occurs, thus dictating the deacetylation pattern. The structural superposition of all available structures of CE4 enzymes active on COS revealed the significance of loop topology as a determinant of such substrate binding specificities (Figure 8), leading to the formulation of the “Subsite Capping Model” [62].

The distinctive α/β barrel of the NodB homology domain is highly conserved. The substrate binding cleft is located on one side of the barrel, where structural variability is notably higher. This variability is provided by a series of loops surrounding the active site that connect the α/β elements of the barrel. There are six loops (Loop 1 to Loop 6) of different length, structure, and sequence composition that modulate the shape of the binding cleft exposed to the substrate in each particular enzyme (Figure 8). Taking as reference the 3D structure of *Vc*CDA in complex with *N,N*′*,N*′′-triacetylchitotriose, these loops define three different substrate binding subsites along the binding cleft of the enzyme (Figure 7). Analogously to the numbering of subsites in glycosyl hydrolases, these are numbered as −n, 0 and +n, from the non-reducing end to the reducing end of the substrate, with subsite 0 being the site of deacetylation (catalytic center) [49]. In *Vc*CDA, the non-reducing *N*-acetylglucosamine unit is placed at subsite −1 (defined by Loops 1, 2, and 6), whereas the reducing *N*-acetyl glucosamine unit is placed at subsite +1 (defined by Loops 3, 4, and 5). No additional binding subsites can be identified in the structure of *Vc*CDA, towards neither the reducing nor the non-reducing ends, that would allow binding of longer substrates. The binding cavity is so tight that substrates cannot slide along the cleft, such as a multiple-attack mechanism would require (Figure 2). Indeed, subsite 0 is exclusively occupied by the *N*-acetylglucosamine unit located at the second position after the non-reducing end. These facts are in agreement with the preferential activity of *Vc*CDA towards short chitin oligosaccharides, and with strict specificity for a single deacetylation event and a unique pattern of deacetylation.

An equivalent distribution of subsites along the substrate binding cleft can be observed in all the structures of CE4 enzymes active on COS (Figure 8). The catalytic site defined by the metal coordination triad is located exactly at the center of this loops bundle (subsite 0), and the accessibility to additional substrate binding subsites is differentially blocked by these loops. On one hand, Loop 1, Loop 2, and Loop 6 contribute to define the negative subsites, where the non-reducing end of the substrate is placed. For instance, Loop 1 and Loop 6 are notably longer in *Vc*CDA and NodB compared to the rest of CDAs. As a consequence, negative subsites are partially hindered, and deacetylation takes place specifically at the non-reducing-end edge of the substrates. On the other hand, Loop 3, Loop 4, and Loop 5 contribute to define the positive subsites, where the reducing end of the substrate is placed. Except in *Vc*CDA, these loops are relatively short in all CDAs, and positive subsites are highly exposed to the solvent and substrates. The scarce existence of CDAs that efficiently deacetylate the reducing end of COS may indicate that binding of a GlcNAc unit at subsite +1 is essential for the enzymatic activity. *Vc*CDA is the only known CE4 enzyme able to exclusively deacetylate the reducing end unit of *N,N*′-diacetylchitobiose. In this enzyme, positive subsites are completely blocked by Loop 4 and the longest Loop 5 in the family, which dynamically assemble the substrate binding pocket (see above). Finally, it is interesting to note that the shuffling point along the sequence of poly-β-1,6-GlcNAc deacetylases takes place within Loop 5.

A second X-ray structure of a CDA in complex with substrate has been recently reported (November 2017), that of *Ar*CE4 from a marine *Arthobacter* species [64]. Even though the enzyme was co-crystallized with a DP4 substrate, only the electron density for a GlcNAc dimer occupying subsite 0 and +1 could be solved. Apparently, the other two rings are not stabilized by any protein–ligand interactions, and they may adopt multiple orientations. *Ar*CE4 has short loops 1 to 6, resulting in an open cleft with only two defined subsites (0 and +1) and allowing binding of longer substrates on a shallow binding cleft. Binding of the sugar in the +1 subsite seems to be dominated by a stacking interaction with a Trp residue. Similarly to *Vc*CDA, the GlcNAc unit bound in subsite 0 is properly oriented for catalysis, and has multiple interactions with the enzyme. *Vc*CDA and *Ar*CE4 deacetylases represent opposite cases with regard to the size of the loops surrounding the active site cleft. *Vc*CDA, with long loops shaping the binding cleft, prefers a short DP2 substrate, and the activity decreases with increasing chain length of the oligosaccharide substrate; Loops 1 and 6 block the accessibility of additional subsites on the non-reducing end site of the deep binding cleft and they dictate the specificity for deacetylation at the penultimate residue from the non-reducing end, whereas Loops 4 and 5 limit productive binding of longer substrates. In contrast, *Ar*CE4, with short loops (the shortest among characterized CE4 enzyme, Figure 4) and an open and shallow binding cleft, shows increasing activity with longer substrates, and follows a multiple-chain mechanism.

Not only do the size and shape of the loops contribute to define the specificity and pattern of acetylation, but their dynamics also participate in this process. Evidence was provided by the *Vc*CDA enzyme, where docking simulations of longer substrates than those co-crystallized with the enzyme (DP2 and DP3, Figure 7) were unable to accommodate the ligands in productive binding modes, because Loop 5 was blocking access to additional positive subsites beyond the already exposed subsite +1 (as seen in the *Vc*CDA·DP3 complex). However, longer oligosaccharides, such as DP4 and DP5 COS, are substrates of the enzyme, although with a 10-fold reduced efficiency [62]. Both molecular dynamic simulations and loops engineering, where mutations are introduced to impact their dynamics, are supportive of the model [154], providing exciting new opportunities to modify and tune deacetylation patterns.

The topology and dynamics of these loops also mediate specific enzyme–substrate interactions that seem to be related to the different substrate specificities of CE4 enzyme subfamilies. Tyr169 (hereafter *Vc*CDA numbering is used as a reference) located in Loop 3 (MT3 motif RxP**Y**) establishes a hydrophobic interaction with the substrate ring located at subsite 0. An aromatic amino acid residue is always present in the reported CE4 enzymes active on COS, but not in MurNAc peptidoglycan deacetylases, which have an arginine at this position. Trp229 and Leu293 (located at subsite 1, L293 being part of the MT5 motif **L**xHD) define a hydrophobic pocket that accommodates the methyl group of the reactive *N*-acetylglucosamine unit at subsite 0, and the methylene unit of the hydroxymethyl group of the substrate GlcNAc located at subsite +1. Trp229 and Leu293 are highly conserved in the family, except for some fungal enzymes and poly-β-1,6-GlcNAc deacetylases. Asp232 (located at subsite +1, the first D of the MT4 motif **D**xxD) establishes a hydrogen bond with the hydroxyl of the hydroxymethyl group of the GlcNAc unit located at subsite +1, as well as a water-mediated hydrogen bond with the catalytic histidine (His295). The polarity of this amino acid is conserved within the family, except for MurNAc peptidoglycan deacetylases, which carry a non-polar alanine amino acid instead, and poly-β-1,6-GlcNAc deacetylases, which have a leucine at that position. Trp238 (located in Loop 4, MT4 motif DxxD(**W**/Y)) establishes a CH–π interaction with the substrate ring located at subsite 0. An aromatic side chain is predominant in this position within the family, with very few exceptions. Phe297 (located in Loop 6) establishes hydrophobic interactions with the substrate ring located at subsite −1. This is a well conserved hydrophobic position in the family, except for poly-β-1,6-GlcNAc deacetylases, which have a polar substituent. Finally, although not strictly conserved, Asn65, Trp67, and His68 (located at the beginning of Loop 1) coordinate a water molecule that is hydrogen-bonded to the hydroxyl group of the hydroxymethyl substituent of the substrate ring located at subsite −1.

The reducing end of the substrate is not deacetylated, or it is the least reactive GlcNAc unit for most CDAs and related CE4 enzymes active on COS. In the 3D structure of the *Ar*CE4·DP2 complex [64], binding of the GlcNAc unit of the substrate in the +1 subsite seems to be dominated by the stacking interaction with a Trp in Motif 4. This aromatic residue is highly conserved (MT4, DxxD(**W**/**Y**), with few exceptions. CE4 enzymes having this aromatic residue seem to prefer a sugar bound in the +1 subsite, and do not deacetylate the reducing end of their substrates, as shown for the *Ar*CE4 enzyme, as well as for *Pes*CDA [89] and *Pc*CDA [95], or the reducing end is the slowest position to be deacetylated, as shown for *Cl*CDA [49] and *An*CDA [12]. On the contrary, *Vc*CDA has the equivalent aromatic residue in a slightly different position after a two-amino acid insertion in the MT4 motif, and it is part of the Loop 4 that moves from an open to a closed conformation upon substrate binding. As a consequence of the induced fit, the Trp residue establishes a stacking interaction with the GlcNAc unit in subsite 0. DP2 is the preferred substrate of *Vc*CDA, and it is deacetylated at the reducing end [62]. Likewise, *Pgt*CDA lacks the +1 aromatic residue, and it deacetylates the reducing end GlcNAc unit of all substrates from DP4 to DP6 [89]. Although these observations seem a general trend, there is still limited information to gain insights into the structural determinants that dictates specificity and deacetylation patterns, and more structural data on enzyme·substrate complexes is required for better understanding the functionality of CE4 enzymes.

## 6. Conclusions

The CE4 family is composed of deacetylases that act on structural polysaccharides, such as chitin and chitosans, peptidoglycans, acetylxylans, and β-1,6-GlcNAc polysaccharides. Here, we have reviewed the characterized enzymes in the family that deacetylate chitin and chitooligosaccharide (COS) substrates. The CE4 family members share an (β/α)_8_ three-dimensional fold, and are metal-depended hydrolases, with Zn^2+^ and Co^2+^ as the most common metal cations. A signature of the family is the conservation of five active site motifs that include the His–His–Asp metal binding triad, and the catalytic Asp and His residues as general base and general acid, respectively. The structural determinants of substrate specificity and deacetylation pattern exhibited by CDAs and related CE4 enzymes active on COS remain poorly understood. The recently solved crystallographic structures of few family members, particularly those of the *Vibrio cholerae* CDA in complex with substrates, have provided some insights into structure-specificity relationships. The substrate binding cleft, located on one side of the distinctive β/α barrel of the NodB homology domain, is shaped by a series of loops that surround the active site. These loops, which differ in length, sequence composition, structure and dynamics, modulate the exposure of the different subsites along the binding cleft to the substrate in each particular enzyme. Long loops at the edges of the binding cleft are found in chitin oligosaccharide deacetylases, such as *Vc*CDA and rhizobial NodB deacetylases, which are specific for a single site deacetylation. Short loops make up a more open binding cleft able to bind chito-oligomers in different manners, as found in CDAs that can catalyze multiple deacetylations on COS substrates, and are active on polymeric substrates. The surface charge distribution along the binding cleft and other structural features may also participate in defining the mode of action and deacetylation pattern exhibited by each particular enzyme. The discovery of novel CDAs with different specificities and the resolution of new enzyme–substrate complex structures will provide further insights into structure-specificity relationships. This knowledge is relevant for the biotechnological applications of CDAs and other CE4 enzymes to design inhibitors targeting CDAs as antimicrobial agents, and to engineer these enzymes as biocatalysts for the production of well-defined partially deacetylated COS with biological activities.

## Figures and Tables

**Table 1:** CE4 enzymes active on chitooligosaccharide and GlcNAc-containing polysaccharides.

Subfamil ^(1)^	Enzyme	Organism	PDB (Year)	Ref ^(2)^	Polymer Substrates	COS Substrates ^(3)^	Metal	PA ^(4)^ (on A_n_)
**Chitin DA**	*Mr*CDA	*Mucor rouxii*	--		Chitin, chitosan	≥DP3	Zn^2+^	D_n,_ D_n−1_A
	*Cl*CDA	*Colletotrichum lindemuthianum*	**2IW0** (2006)	[61]	Glycol-chitin	DP6>DP5>DP4>DP3>DP2	Co^2+^ Zn^2+^	D_n_
	*An*CDA	*Aspergillus nidulans*	**2Y8U** (2012)	[12]	Glycol-chitin, chitin, CM-chitin, acetylxylan	DP2>DP3>DP4>DP5	Co^2+^	D_n_
	*Pgt*CDA	*Puccinia graminis*	--		Glycol-chitin, colloidal chitin, chitosans	DP6>DP5>DP4	n.r. ^(5)^	AAD_n−2_
	*Pes*CDA	*Pestolotiopsis* sp.	--		Colloidal chitin, chitosan DA10-60%	DP6-DP5-DP4	n.r.	AAD_n−3_A
	*Pa*CDA	*Podospora anserina*	--		Glycol-chitin	≥DP2	Zn^2+^	D_n_
	*Pc*CDA	*Pochonia chlamydosporia*	--		n.r.	DP5>DP4	n.r.	ADDA_n−3_
	NodB	*Sinorhizobium meliloti*	--		COS	DP5>DP2 (DP4, DP3)	Mn^2+^ Mg^2+^	DA_n−1_
	*Vc*CDA (COD)	*Vibrio cholera*	**4NY2** (2014)	[62]	COS	DP2>DP3>DP4>DP5>DP6	Zn^2+^	ADA_n−2_
	*Vp*CDA (COD)	*Vibrio parahaemolyticus*	**3WX7** (2014)	[63]	COS	DP2, DP3	Zn^2+^	n.r.
	*Ar*CE4	*Arthrobacter* sp.	**5LFZ** (2017)	[64]	Chitin, chitosan, acetylxylan	DP5>DP6=DP4>DP3>>DP2	Ni^2+ (6)^	A3D2
**GlcNAc DA**	*Sp*PgdA	*Streptococcus pneumoniae*	**2C1G** (2005)	[65]	GlcNAc DA on peptido-glycan	(GlcNAc)_3_	Zn^2+^	ADA
	*Sm*PgdA	*Streptococcus mutants*	**2W3Z** (2008)	[66]	GlcNAc DA on peptidoglycan	DP6	Zn^2+^	n.r.
	*Bc*Pgd (BC1960)	*Bacillus cereus*	**4L1G** (2014)	[67]	GlcNAc DA on peptido-glycan, glycol-chitin	DP6-DP5-DP4>>DP3>DP2	Co^2+^	D_n−1_A
	*Er*Pgd	*Eubacterium rectale*	**5JMU** (2016)		GlcNAc deacetylase (annotated)		Zn^2+^	
**MurNAc DA**	*Bs*PdaA	*Bacillus subtilis*	**1W17** (2005)	[68]	MurNAc DA on peptido-glycan (Cwld digested)	No active on COS	Cd^2+ (6)^	
	*Ba*Pda (BA0424)	*Bacillus anthracis*	**2J13** (2006)	[69]	MurNAc DA on peptido-glycan	n.r.	Zn^2+^	
**PPda (unk)**	BC0361	*Bacillus cereus*	**4HD5** (2012)	[70]	Substrate unknown Putative GlcNAc DA		Zn^2+^	
	BA0330	*Bacillus anthracis*	**4V33** (2015)	[71]	Unknown. Not active on glycol-chitin, COS, pNPAc, synthetic muropeptide		Zn^2+^	
	BA0150	*Bacillus anthracis*	**4M1B** (2014)	[72]	Presumably inactive (no metal coordination)		No metal	
	ECU11_0510	*Encephalitozoon cuniculi*	**2VYO** (2009)	[73]	Inactive (lack of Asp general base and His metal-binding)		No metal	
**AXE**	*Sl*AxeA	*Streptomyces lividans*	**2CC0** (2006)	[74]	Acetylxylan, glycol-chitin, chitosan	DP2-DP4-DP6	Co^2+^	DDD (A1D2)
	*Ct*AxeA	*Clostridium thermocellum*	**2C71** (2006)	[74]	2-*O*-acetylxylan	No active on COS	Co^2+^	
**β-1,6-GlcNAc DA**	*Ec*PgaB	*Escherichia coli*	**3VUS** (2012)	[75,76]	Poly-β-1,6-GlcNAc de-*N*-acetylase	β-1,6-GlcNAc oligomers	Co^2+^ Ni^2+^ Zn^2+^	
	*Ad*IcaB	*Ammonifex degensii*	**4WCJ** (2014)	[77]	Poly-β-1,6-GlcNAc de-*N*-acetylase	β-1,6-GlcNAc oligomers	Ni^2+^ Co^2+^ Zn^2+^	
	*Bb*BpsB	*Bordetella bronchiseptica*	**5BU6** (2015)	[45]	Poly-β-1,6-GlcNAc de-*N*-acetylase	β-1,6-GlcNAc oligomers	Ni^2+^ Co^2+^	
	*Aa*PgaB	*Aggregatibacter actinomycetemcomitans*	**4U10** (2015)		Poly-β-1,6-GlcNAc de-*N*-acetylase	β-1,6-GlcNAc oligomers	Zn^2+^	

^(1)^ Chitin DA: chitin deacetylase; GlcNAc DA: peptidoglycan *N*-acetylglucosamine deacetylase; MurNAc DA: peptidoglycan *N*-acetylmuramic deacetylase; PPda (unk): putative polysaccharide deacetylase (unknown); AXE: acetylxylan esterase; β-1,6-GlcNAc DA: poly-β-1,6-*N*-acetylglucosamine deacetylase. ^(2)^ 3D structure publication. ^(3)^ Activity on chitooligo-saccharides (β-1,4-linked GlcNAc oligomers) as a function of the degree of polymerization (DP). ^(4)^ Pattern of acetylation (PA). Structure of the main final deacetylated product. A: GlcNAc, D: GlcNH_2_. Other patterns of acetylation with specific substrates are given in the text. ^(5)^ n.r.: not reported. ^(6)^ No evidence for native metal, but indicated the metal from purification/crystallization experiments.

**Table 2:** Modular organization of domains in CE4 enzymes. Modular structures were defined using the Interpro database tools [149].

Enzyme	Uniprot AC	# aa FL ^1^	CE4 (aa) ^2^	Modular Structure
*Mr*CDA	CDA_AMYRO	421	151–349	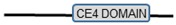
*Cl*CDA	Q6DWK3_COLLN	248	30–246	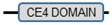
*An*CDA	B3VD85_EMEND	249	44–245	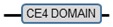
*Pgt*CDA	E3K3D7_PUCGT	269	38–236	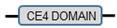
*Pes*CDA	A0A1L3THR9_9PEZI	298	27–236	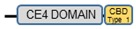
*Pa*CDA	B2AAQ0_PODAN	396	120–307	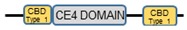
*Pc*CDA	--	455	107–303	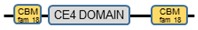
NodB	NODB_RHIME	217	15–213	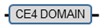
*Vc*CDA	Q9KSH6_VIBCH	431	26–338	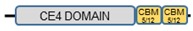
*Vp*CDA	A6P4T5_VIBPH	427	28–326	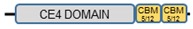
*Ar*CE4	A0A2C8C1T7_9MICC	246	42–227	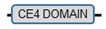
*Sp*PgdA	Q8DP63_STRR6	463	264–454	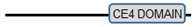
*Sm*PgdA	Q8DV82_STRMU	311	103–308	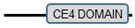
*Bc*Pgd BC1960	B9J460_BACCQ	275	68–266	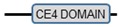
*Er*Pgd	C4ZEZ9_AGARV	496	290–482	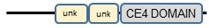
*Bs*PdaA	PDAA_BACSU	266	68–253	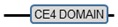
*Ba*Pda	Q81Z49_BACAN	260	45–255	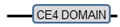
BC0361	Q81IM3_BACCR	360	195–360	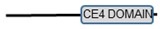
BA0330	Q81ZD9_BACAN	360	195–360	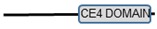
BA0150	Q81VP2_BACAN	254	52–237	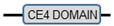
ECU11_	YB51_ENCCU	254	26–210	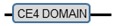
*Sl*AxeA	Q54413_STRLI	335	44–221	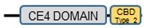
*Ct*Axe (*XynA*)	O87119_CLOTM	683	477–655	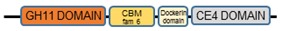
*Ec*PgaB	PGAB_ECOLI	672	65–349	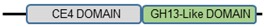
*Ad*IcaB	C9RCK9_AMMDK	280	67–280	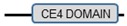
*Bb*BpsB	A0A058YIS5_BORBO	701	66–355	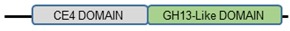
*Aa*PgaB	A5HJW8_AGGAC	638	48–334	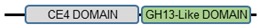

^1^ Total number of amino acid residues in the full length protein. ^2^ Amino acid numbering for the CE4 catalytic domain.

## Abbreviations

AXE	Acetylxylan estarase
A_n_	(GlcNAc)_n_
CDA	Chitin deacetylase
COS	Chitooligosaccharides
D_n_	(GlcNH_2_)_n_
DA	Degree of acetylation
DP	Degree of polymerization
GlcNAc	*N*-acetylglucosamine
GlcNH_2_	Glucosamine
MurNAc	*N*-acetylmuramic acid
PA	Pattern of acetylation
PDB	Protein data bank
PG	Peptidoglycan
PNAG	Poly-β-1,6-*N*-acetyl-d-glucosamine
PPda	Putative polysaccharide deacetylase
